# Supplementary material for: The Effects of Short-Term PM2.5 Exposure on Pulmonary Function among Children with Asthma—A Panel Study in Shanghai, China
Source: Int J Environ Res Public Health. 2022 Sep 9;19(18):11385. doi: 10.3390/ijerph191811385 (PMC9517124; doi:10.3390/ijerph191811385)
Supplement: Supplementary file 1 [file ijerph-19-11385-s001.zip › ijerph-1873999-supplementary.pdf]

Supplementary Material

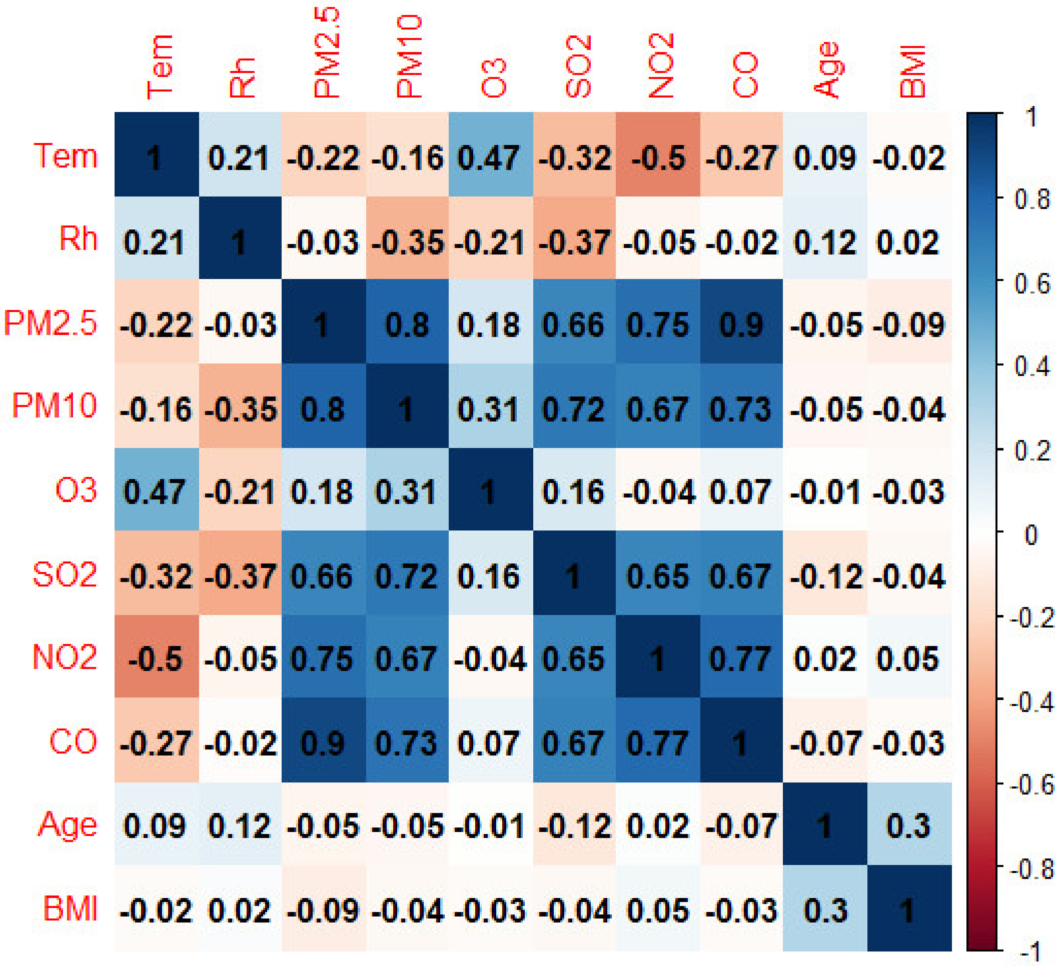

	Tem	Rh	PM2.5	PM10	O3	SO2	NO2	CO	Age	BMI
Tem	1	0.21	-0.22	-0.16	0.47	-0.32	-0.5	-0.27	0.09	-0.02
Rh	0.21	1	-0.03	-0.35	-0.21	-0.37	-0.05	-0.02	0.12	0.02
PM2.5	-0.22	-0.03	1	0.8	0.18	0.66	0.75	0.9	-0.05	-0.09
PM10	-0.16	-0.35	0.8	1	0.31	0.72	0.67	0.73	-0.05	-0.04
O3	0.47	-0.21	0.18	0.31	1	0.16	-0.04	0.07	-0.01	-0.03
SO2	-0.32	-0.37	0.66	0.72	0.16	1	0.65	0.67	-0.12	-0.04
NO2	-0.5	-0.05	0.75	0.67	-0.04	0.65	1	0.77	0.02	0.05
CO	-0.27	-0.02	0.9	0.73	0.07	0.67	0.77	1	-0.07	-0.03
Age	0.09	0.12	-0.05	-0.05	-0.01	-0.12	0.02	-0.07	1	0.3
BMI	-0.02	0.02	-0.09	-0.04	-0.03	-0.04	0.05	-0.03	0.3	1

Figure. S1. Pearson's correlation coefficients between factors affecting lung function. Tem, Daily temperature; Rh, Relative humidity; $PM_{2.5}$, fine particulate matter with aerodynamic diameter ≤ 2.5 μm; PM_{10}, particulate matter with aerodynamic diameter ≤ 10 μm; O_3, ozone; SO_2, sulfur dioxide; NO_2, nitrogen dioxide; CO, carbon monoxide.

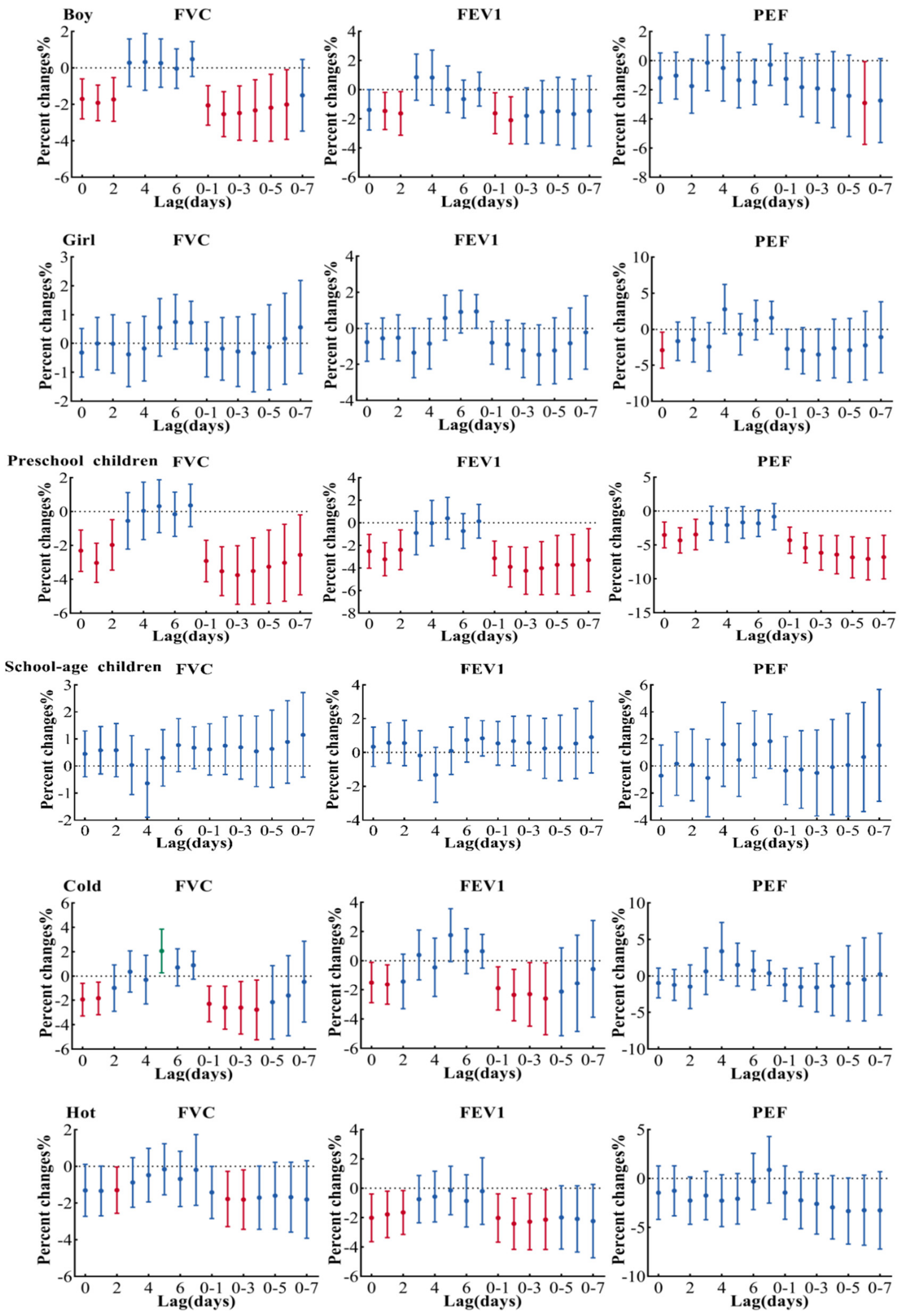

Figure. S2. Association between each lung function index and $PM_{2.5}$ in the stratified analysis of $PM_{2.5}$ single pollution model. Preschool children : Age < 6 years old; School-age children: age ≥ 6 years old. Cold season refers to the period of Nov to Mar; Hot season means May to Sept. Estimates are adjusted for questionnaire information, relative humidity, temperature, age, BMI, sex, holiday, day of week and times of the measurement. FVC, forced vital capacity; FEV1, forced expiratory volume in 1 second; PEF, peak expiratory flow.

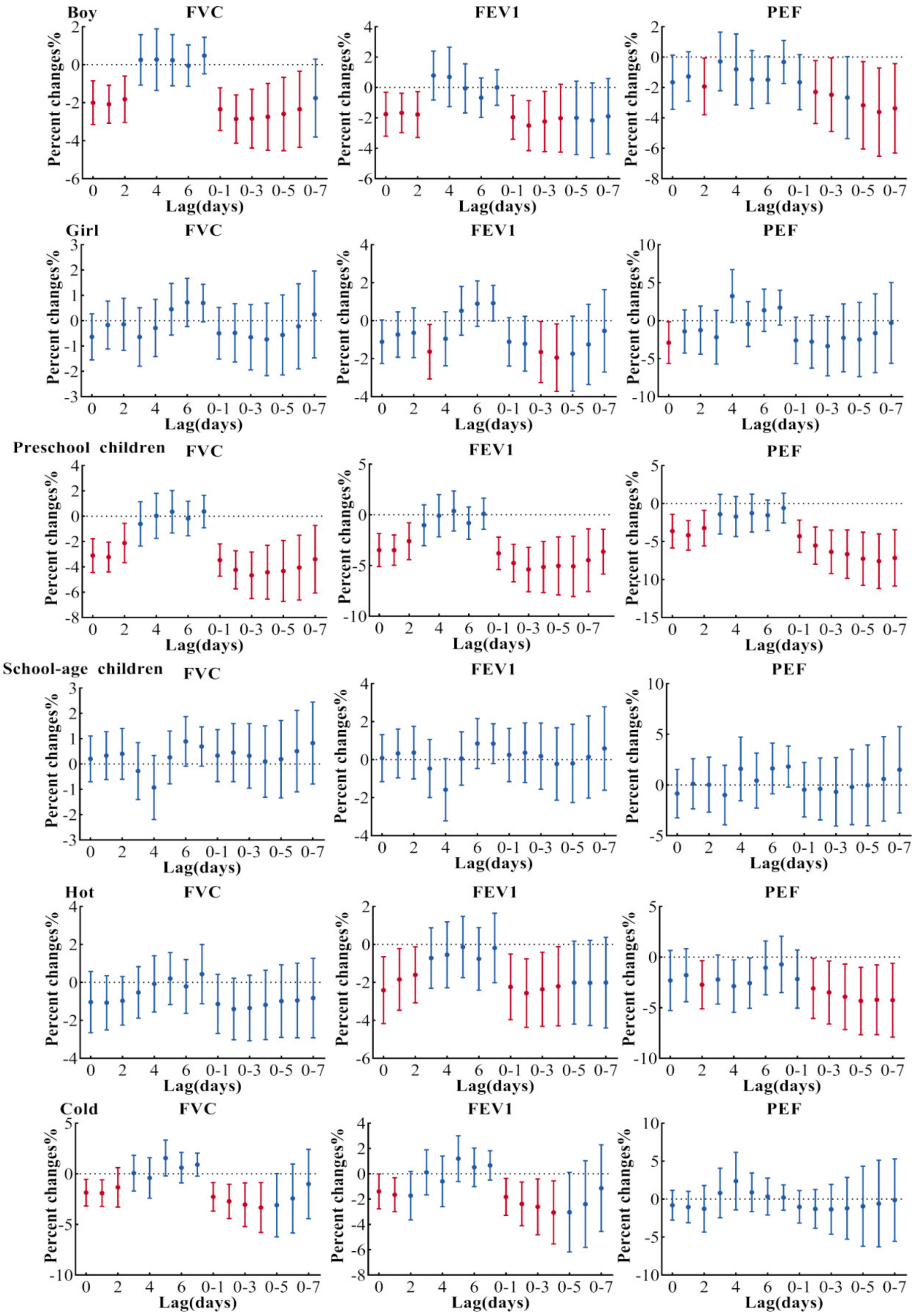

Figure. S3. Association between each lung function index and $PM_{2.5}$ in a stratified analysis of a two-pollution model (adjusted $PM_{2.5}$+O_3). Preschool children: Age < 6 years old; School-age children: age ≥ 6 years old. Cold season refers to the period of Nov to Mar; Hot season means May to Sept. Estimates are adjusted for questionnaire information, relative humidity, temperature, age, BMI, sex, holiday, day of week and times of the measurement. FVC, forced vital capacity; FEV1, forced expiratory volume in 1 second; PEF, peak expiratory flow.

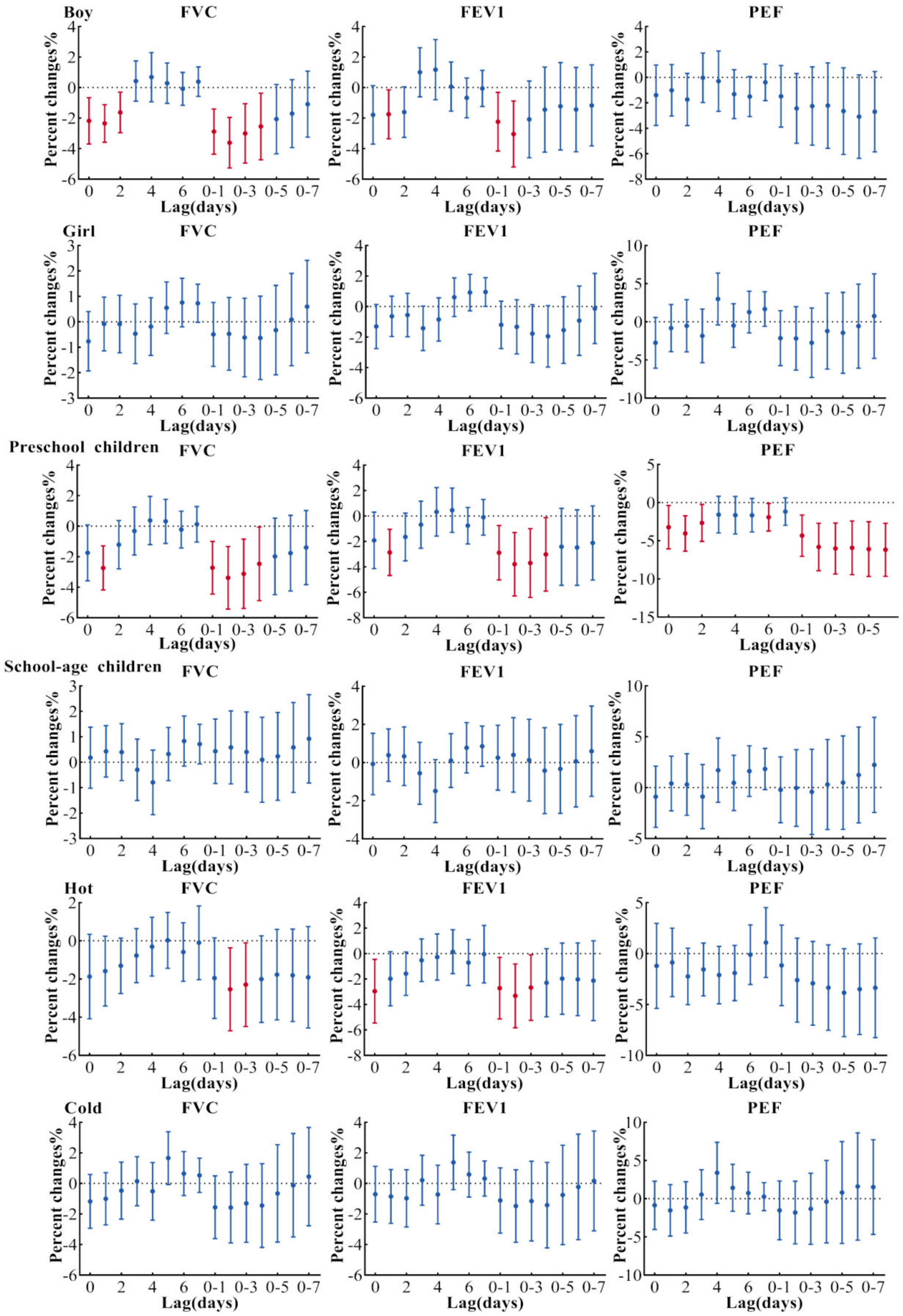

Figure. S4. Association between each lung function index and $PM_{2.5}$ in a stratified analysis of a two-pollution model (adjusted $PM_{2.5}$+SO_2). Preschool children: Age < 6 years old; School-age children: age ≥ 6 years old. Cold season refers to the period of Nov to Mar; Hot season means May to Sept. Estimates are adjusted for questionnaire information, relative humidity, temperature, age, BMI, sex, holiday, day of week and times of the measurement. FVC, forced vital capacity; FEV1, forced expiratory volume in 1 second; PEF, peak expiratory flow.

Questionnaire

Basic demographic information

1.Name:____________

2.Gender: ☐Boy ☐Girl

3.Age:___________

4.Date of birth:_____________

5.Weight:________kg

6.High:________cm

7.Date of visit:_________

Asthma related information

1.Does your child have eczema/ringworm, or a doctor-diagnosed atopic dermatitis?

☐Yes ☐No

2.Does your child have frequently nasal congestion, itching, runny nose, sneezing, or a doctor-diagnosed allergic rhinitis?

☐Yes ☐No

3.Does your child have an established food or drug allergy?

☐Yes ☐No

4.Dose the child's mother had any of the following allergic diseases?

☐Atopic dermatitis or urticaria ☐Allergic rhinitis ☐Asthma ☐No

5.Dose the child's father had any of the following allergic diseases?

☐Atopic dermatitis or urticaria ☐Allergic rhinitis ☐Asthma ☐No

6.Dose anyone frequently smoked in places where your child often had activities or rest? The frequency is:

☐No ☐ < 1 unit/day ☐1~5 unit/day ☐> 5 unit/day

7.Dose any smoker come into contact with your child (regardless of where the smoker was smoking)? frequency is

☐No ☐ < 1 h/day ☐1~4 h/day ☐4~8 h/day ☐> 8 h/day
